# Normal Human Pluripotent Stem Cell Lines Exhibit Pervasive Mosaic Aneuploidy

**DOI:** 10.1371/journal.pone.0023018

**Published:** 2011-08-16

**Authors:** Suzanne E. Peterson, Jurjen W. Westra, Stevens K. Rehen, Holly Young, Diane M. Bushman, Christine M. Paczkowski, Yun C. Yung, Candace L. Lynch, Ha T. Tran, Kyle S. Nickey, Yu-Chieh Wang, Louise C. Laurent, Jeanne F. Loring, Melissa K. Carpenter, Jerold Chun

**Affiliations:** 1 Department of Molecular Biology, Dorris Neuroscience Center, The Scripps Research Institute, La Jolla, California, United States of America; 2 Department of Chemical Physiology, The Scripps Research Institute, La Jolla, California, United States of America; 3 Biomedical Sciences Graduate Program, School of Medicine, University of California San Diego, San Diego, California, United States of America; 4 Institute of Biomedical Sciences, Federal University of Rio de Janeiro, Rio de Janeiro, Brazil; 5 Robarts Research Institute, London, Ontario, Canada; 6 Department of Reproductive Medicine, University of California San Diego, San Diego, California, United States of America; University of Minnesota, United States of America

## Abstract

Human pluripotent stem cell (hPSC) lines have been considered to be homogeneously euploid. Here we report that normal hPSC – including induced pluripotent - lines are karyotypic mosaics of euploid cells intermixed with many cells showing non-clonal aneuploidies as identified by chromosome counting, spectral karyotyping (SKY) and fluorescent *in situ* hybridization (FISH) of interphase/non-mitotic cells. This mosaic aneuploidy resembles that observed in progenitor cells of the developing brain and preimplantation embryos, suggesting that it is a normal, rather than pathological, feature of stem cell lines. The karyotypic heterogeneity generated by mosaic aneuploidy may contribute to the reported functional and phenotypic heterogeneity of hPSCs lines, as well as their therapeutic efficacy and safety following transplantation.

## Introduction

The generation of human embryonic stem cells (hESCs) initiated a promising new area of scientific research [1]. With the advent of induced pluripotent stem cells (iPSCs) and continued research into hESCs, we have learned much about pluripotency [2], [3], [4], [5], [6], [7], [8]. Although human pluripotent stem cells (hPSCs) have been successfully used to treat mouse models of many diseases and are even currently being used in four Phase 1 clinical trials worldwide [9], there is much that remains unknown about these cells. An emerging theme in stem cell biology is that hPSCs are not homogeneous, instead showing phenotypic and functional heterogeneity within cultures. This heterogeneity can be seen in differences in marker expression, functionality and epigenetic patterns [10], [11], [12], [13], [14], [15], [16]. Indeed, the notion of hPSCs as having binary fates – either totally pluripotent or totally differentiated has come into serious question. Rather, it appears that hPSCs exist on a continuum between pluripotent and differentiated states [10], [11], [12].

The mechanisms that generate heterogeneity within hPSC cultures are unknown but processes including “transcriptional noise” and epigenetic heterogeneity have been suggested [11], [12], [17]. In regard to other mechanisms that may contribute to heterogeneity, recent findings in the normal developing and mature vertebrate brain indicate that many progenitor and fate-committed cells are not euploid but are instead mosaically aneuploid – showing myriad karyotypic differences consisting of non-clonal chromosomal gains and/or losses [18], [19], [20], [21], [22]. Indeed, in the developing brain approximately 1/3 of the cells show mosaic aneuploidy [18], [20]. These aneuploid neural stem cells do not simply die but instead differentiate into mature, functional neurons [18], [19], [23]. In addition, karyotypic differences among neural stem cells likely affect cell function by generating changes in gene expression [24]. Thus, karyotypic heterogeneity in neural cells leads to heterogeneity in gene expression and presumably cell function.

If mosaic aneuploidy exists normally in hPSC populations, such karyotypic heterogeneity could potentially contribute to the phenotypic and functional heterogeneity recently described in hPSC cultures [10], [11], [12], [13], [14], [15], [16]. Consistent with this idea, cells from the inner cell mass – the cells that are used to create hESC lines – also exhibit mosaic aneuploidy [25], [26], [27], [28]. Preimplantation genetic screening (PGS) has been used extensively to diagnose genetic diseases in IVF embryos. In this procedure, a single blastomere is removed from a 3-day embryo and FISH is done to interrogate the presence of 4–6 individual chromosomes. Though PGS is based on the idea that analysis of a single blastomere will be representative of the entire embryo, this has been repeatedly proven incorrect [25], [26], [27], [29]. When multiple blastomeres from the same blastocyst are analyzed they are often karyotypically non-identical indicating that the blastocyst is chromosomally mosaic [30], [31].

Despite the fact that mosaic aneuploidy is seen in cells from the inner cell mass as well as other types of stem cells including neural progenitor cells, normal hPSCs have been defined as homogenously euploid in publications as well as in a dominant, existing patent [1],[32],[33]. It is notable that aneuploidy in neural progenitor cells is most frequently mosaic hypoploidy produced by chromosomal loss and identified in both mitotic and non-mitotic cells [18]. Since standard cytogenetic texts consider hypoploidy to be an artifact arising from chromosomes “floating away” during metaphase chromosome spread preparation [34], [35], [36], mosaically aneuploid hPSCs may have been previously dismissed as an artifact. The alternative explanation is that mosaic aneuploidy represents a previously unrecognized, normal characteristic of hPSC lines, which may contribute to their phenotypic and functional heterogeneity. Here we have analyzed multiple hPSC lines for mosaic aneuploidy using three independent techniques to visualize chromosome gain and/or loss. We found that all hPSC lines analyzed exhibit pervasive mosaic aneuploidy ranging from ∼18–35% of cells within the culture, indicating that this is a fundamental feature of normal hPSC lines.

## Materials and Methods

### Cell culture

hPSC lines analyzed included WA01, WA07, WA09, and WA14 (also known as H1, H7, H9 and H14) [1], BG01s [37], Cythera25 [38] and HDF6iPS3 (derived in the Loring lab under UCSD SCRO #E08-002 and Scripps Health IRB protocol #HSC-07-4906). Fibroblasts used to generate HDF6iPS3 were collected with appropriate written informed consent. hPSC lines were cultured by 6 different individuals in 4 different labs. Data were analyzed by 3 different individuals. Culture conditions are listed in table 1. Medium was changed every day on all lines except H14, which had medium changes every other day. Plates were coated with 0.1% gelatin before seeding MEFs or Hs27. MEFs were seeded at 1.2×10^6^ cells per 6 well plate. Hs27 were seeded at 1.2×10^6^ cells per 6 well plate. Matrigel (BD Biosciences) was diluted to 1∶200 in HBSS or DMEM. Collagenase was used at 1 mg/ml, while trypsin was 0.05%. bFGF was purchased from Invitrogen, Chemicon or Stemgent at concentrations ranging from 4 ng/mL to 20 ng/mL (see table 1). Non-essential amino acids were used at 1× as was Glutamax. β-mercaptoethanol was used at 0.1 mM. For StemPro medium, the BSA supplement is added as well as 0.1 mM β-mercaptoethanol. All medium, supplements, and passaging enzymes were purchased from Invitrogen. Metaphase spreads and nuclei isolated from human lymphocytes were prepared according to standard methods in accordance with Scripps Health IRB protocol #HSC-00-2105 [19], [34]. Lymphocytes were collected with appropriate written informed consent.

**Table 1:** Culture conditions.

Cell line	Medium	Supplements	Substrate	[bFGF]	passaging
H1 p40	KODMEM	20% KOSR	MEFs	4 ng/ml	Trypsin
Cythera25 p53	DMEM/F-12	20% KOSR	MEFs	4 ng/ml	Mechanical
H14 p45	DMEM/F-12	20% KOSR	MEFs	20 ng/ml	Mechanical
H9 p37	DMEM/F-12	20% KOSR	MEFs	4 ng/ml	Collagenase
H7 p43	DMEM/F-12	20% KOSR	MEFs	4 ng/ml	Collagenase
BG01 p51	DMEM/F-12	StemPro	Matrigel	8 ng/ml	Accutase
H7 p51–64	DMEM/F-12	20% KOSR	Hs27	20 ng/ml	Mechanical
H9 p46–68	DMEM/F-12	20% KOSR	Hs27	20 ng/ml	Mechanical
HDF6iPS3 p17	DMEM/F-12	20% KOSR	MEFs	12 ng/ml	Mechanical

hPSC culture conditions are diverse. No single culture variable including medium, supplements, substrate, bFGF concentration, or passaging technique is consistent among hPSC lines showing mosaic aneuploidy. This suggests that mosaic aneuploidy is not caused by particular culture conditions. In addition, the hPSC cell lines were cultured by 6 different individuals, further suggesting that mosaic aneuploidy is not an artifact related to cell culture.

### Chromosome counts and Spectral Karyotyping

hPSC cultures were processed for chromosome spread preparation according to published methods [18], [19], [34], [39]. For chromosome counts, metaphase chromosome spreads were stained with 4′,6-diamidino-2-phenylindole (DAPI) and counted by fluorescence microscopy. Software from Applied Spectral Imaging was used to aid in chromosome counting. For each cell line, at least 100 metaphase chromosome spreads were counted. SKY was preformed following the manufacturer's instructions (Applied Spectral Imaging) [40]. Forty metaphase spreads were analyzed by SKY per cell line.

### Fluorescent in situ hybridization (FISH)

Whole cells were fixed in 3∶1 methanol: glacial acetic acid at 4°C and then affixed to glass slides. Slides were then pretreated with 50 µg/ml pepsin in 0.01 M HCl for 5 minutes at 37°C. Next, slides were incubated with 50 mM MgCl_2_ in PBS for 5 minutes then in the same solution containing 1% formaldehyde for 10 minutes. Slides were then dehydrated and stored in a desiccator at −20°C until use. FISH probes were generated using FISH Tag™ kits for Alexa Fluor 488 and 555 according to the manufacturer's instructions (Molecular Probes, Eugene, OR). Template DNA used for nick translation was obtained from BACs containing sequences from human chromosome 21 and human chromosome 4 (Children's Hospital Oakland Research Institute). Error rates for these dual red/green FISH probes were determined to be less than 0.01% using interphase lymphocytes and chromosome paints. FISH probes were denatured at 80°C for 10 minutes then reannealed at 37°C for 60 minutes. Probes were then applied to the slide on a coverslip, sealed with rubber cement, and hybridized overnight at 37°C. The next day slides were washed at 45°C for 5 min each in 2XSSC with 50% formamide pH 7.0, 1XSSC, and 2XSSC with 0.1% tween-20. Finally, slides were stained with DAPI (0.3 µg/ml), dehydrated and mounted with a coverslip and vectashield (Vector labs). Between 3000 and 3500 nuclei were analyzed per sample.

## Results

To address the possibility that hPSCs may exhibit chromosomal mosaicism, multiple hPSC lines were examined for mosaic aneuploidy using three distinct and independent techniques. First, hPSC lines were assessed using basic chromosome counting where the number of chromosomes in individual metaphase chromosome spread is quantified using 4′,6-diamidino-2-phenylindole (DAPI) staining and fluorescence microscopy. Second, specific karyotypes were determined using spectral karyotyping (SKY), where each individual chromosome is “painted” a spectrally distinct color. Third, FISH, utilizing independently synthesized point probes for specific chromosomes, was used to assess the chromosomal content of interphase or non-mitotic cells. Importantly, FISH analysis utilized intact interphase nuclei and therefore was not subject to hypothetical artifacts associated with metaphase chromosome spreads.

### All examined hPSC lines grown in different culture conditions by different investigators exhibit pervasive mosaic aneuploidy

**Figure 1:**
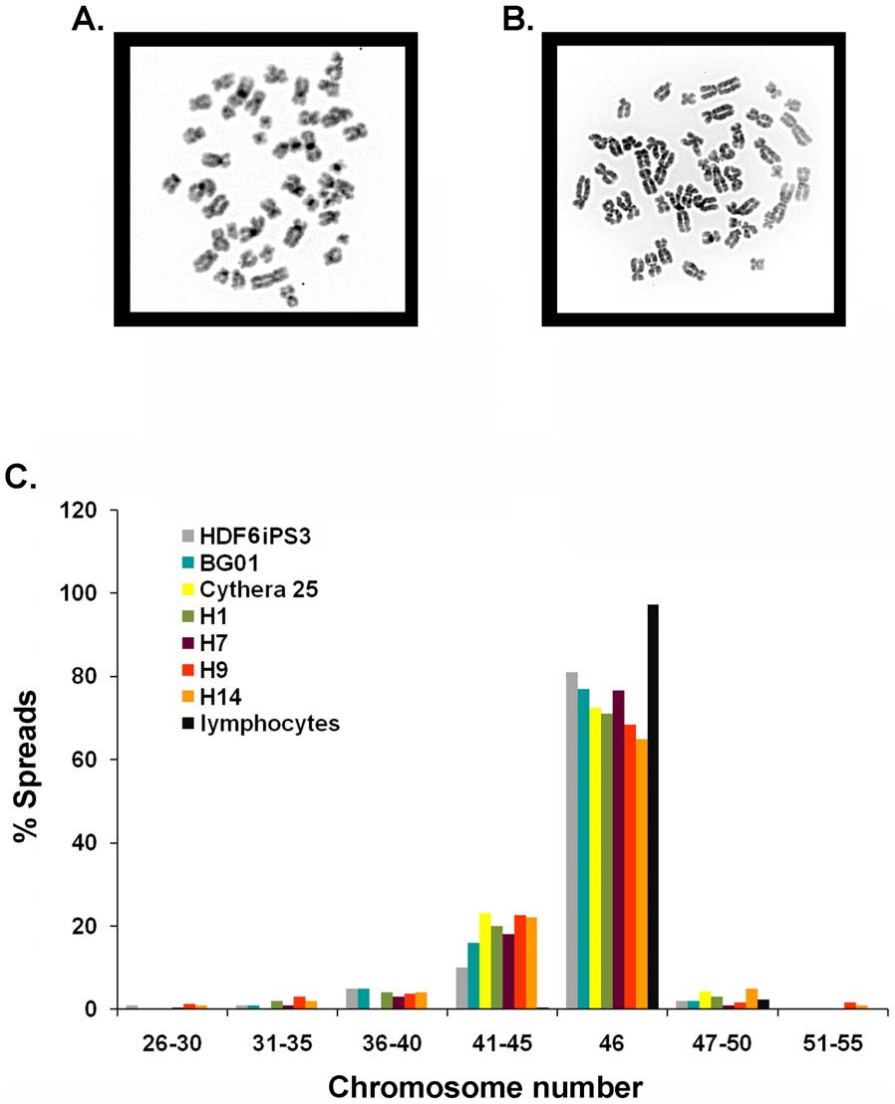
hPSC lines contain significant levels of mosaic aneuploidy that are not seen in human lymphocytes. Metaphase chromosome spreads from H1, H7, H9, H14, Cythera25, BG01, HDF6iPS3 and human lymphocytes were stained with DAPI and chromosome numbers were counted. A representative putatively euploid metaphase spread with 46 chromosomes is shown (A) as well as an aneuploid metaphase chromosome spread with 48 chromosomes (B). C) Each metaphase spread was categorized into a bin (i.e spreads with chromosome numbers 26–30, 31–35, 36–40, 41–45, 46, 47–50 and 51–55) based on how many chromosomes it contained with chromosome numbers for each bin indicated along the x axis in the figure. Each hPSC line showed significant levels of mosaic aneuploidy ranging from 18–35%, while lymphocytes showed very low levels of aneuploidy (<3%).

To determine whether hPSC lines were aneuploid mosaics, we analyzed four well-characterized WiCell lines WA01, WA07, WA09, and WA14 (also known as H1, H7, H9 and H14), all of which have been reported as 100% euploid prior to culture adaptation [1], [37], [41], [42], [43]. The cells were from relatively early passages (H1 passage 40, H7 passage 43, H9 passage 37, and H14 passage 45) and were cultured according to standard protocols by multiple investigators [1]. Note that the earliest passages commercially available for WA01, WA07, WA09, and WA14 are p31, p22, p25, and p20, respectively (http://www.wicell.org/index.php?option=com_oscommerce&Itemid=192). In addition, several non-WiCell lines including BG01 [37], Cythera25 [38] and an iPSC line called HDF6iPS3 were similarly cultured by different investigators in different labs. BG01s were from passage 51 (earliest passage commercially available is p37) while Cythera25 were passage 53. The HDF6iPS3 were derived in the lab of Jeanne Loring using the Yamanaka vectors and were at passage 17 at the time of analysis. Derived iPSC lines have also been reported to be 100% euploid [2], [43], [44]. Cells were arrested in metaphase and chromosome spreads were produced according to standard protocols [34]. As a control, normal human lymphocytes were analyzed in parallel, as these cells have been previously reported to be ∼97% euploid and are typically used for cytogenetic analysis [19]. At least one hundred spreads (typically 300 spreads per hPSC line) were analyzed by three independent observers, and aneuploidies were documented along with euploid populations. A euploid, DAPI-stained H1 metaphase chromosome spread (Figure 1A) with 46 chromosomes contrasts with a hyperploid metaphase chromosome spread possessing 48 chromosomes (Figure 1B). Note that the chromosomes in each spread were confined to a tight circle without any trailing chromosomes, indicating that the spreads were intact; realtime visualization of produced metaphase spreads has demonstrated that aneuploidy was inherent to a cell rather than artifactually produced (D. Bushman, A. Mosely & J. Chun, unpublished). Only spreads with this type of morphology were included in analyses.

Remarkably, all seven hPSC lines contained significant levels of mosaic aneuploidy. The percentage of aneuploid cells ranged from ∼18% in HDF6iPS3 cells to as much as 35% in H14 cells (Figure 1C). In contrast, ∼3% mosaic aneuploidy was observed in the lymphocyte control sample. The presence of mosaic aneuploidy was independent of culture conditions since the hPSC lines were cultured by 6 different investigators. Mosaic aneuploidy was identified regardless of culture conditions, including differences in medium, supplement, substrate, bFGF concentration, or passaging technique (Table 1). All examined hPSC lines were derived from different sources, cultured under different conditions, yet universally exhibited mosaic aneuploidy. By contrast, 100% euploidy was never observed in any hPSC line.

### Mosaic aneuploidy persists over time in culture

**Figure 2:**
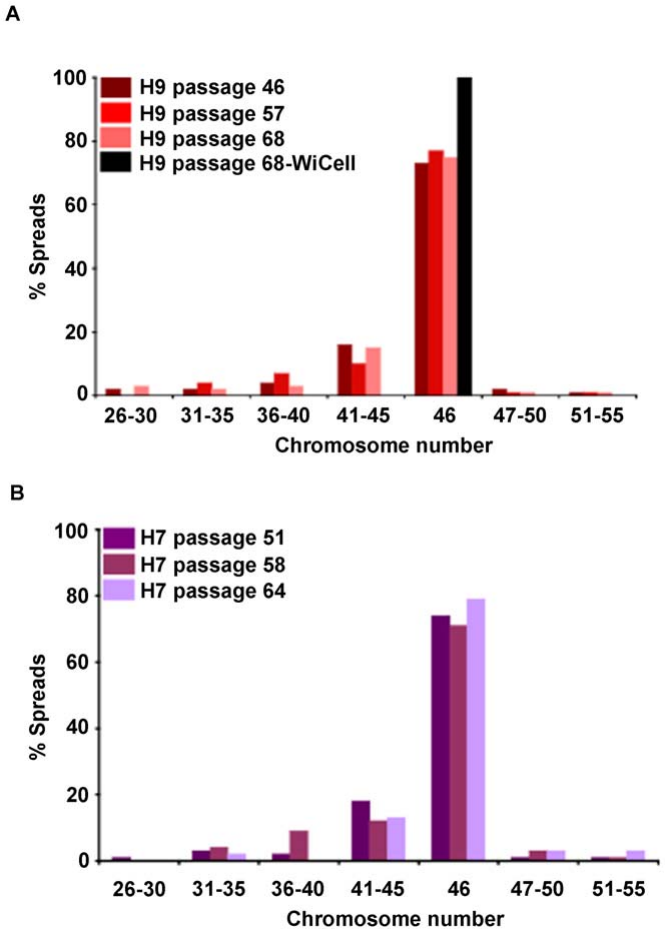
Mosaic aneuploidy exists over time in culture. H9 (A) and H7 (B) were cultured for several passages and analyzed periodically for mosaic aneuploidy via chromosome counts. Specifically, H9 was cultured from passage 46 to 68 and analyzed at passage 46, 57 and 68. H7 was cultured from passage 51 to 64 and analyzed at passage 51, 58 and 64. Both lines show significant levels of mosaic aneuploidy at each of the passages suggesting that mosaic aneuploidy persists with time in culture. Interestingly, H9s at passage 68 were sent out for karyotyping at WiCell and they were deemed 46, XX with no abnormalities detected (black bar).

One potential scenario is that mosaic aneuploidy presents at a particular passage as previously described [45], [46] but then dissipates with time in culture. To investigate this, H7 and H9 lines were cultured for between 13–22 passages and analyzed periodically for chromosome number at 3 different time points (Figure 2 A & B). Specifically, H9 was cultured from passage 46 to passage 68 and analyzed at passage 46, 57 and 68 (Figure 2A). H7 was cultured from passage 51 to 64 and analyzed at passage 51, 58, and 64 (Figure 2B). Consistent with the idea that mosaic aneuploidy is a stable characteristic of hPSC lines, the percentage of mosaic aneuploid cells in H9 cultures was 27, 23 and 25% at the passages analyzed and H7 was 26, 27 and 21% mosaic aneuploid. Thus, mosaic aneuploidy persisted with time in culture and was not passage-dependent. In contrast, H9 at passage 68 was found to be “46, XX with no abnormalities detected” by the WiCell cytogenic facility (supplemental Figure S1).

### Chromosome gain and/or loss appears to be stochastic

**Figure 3:**
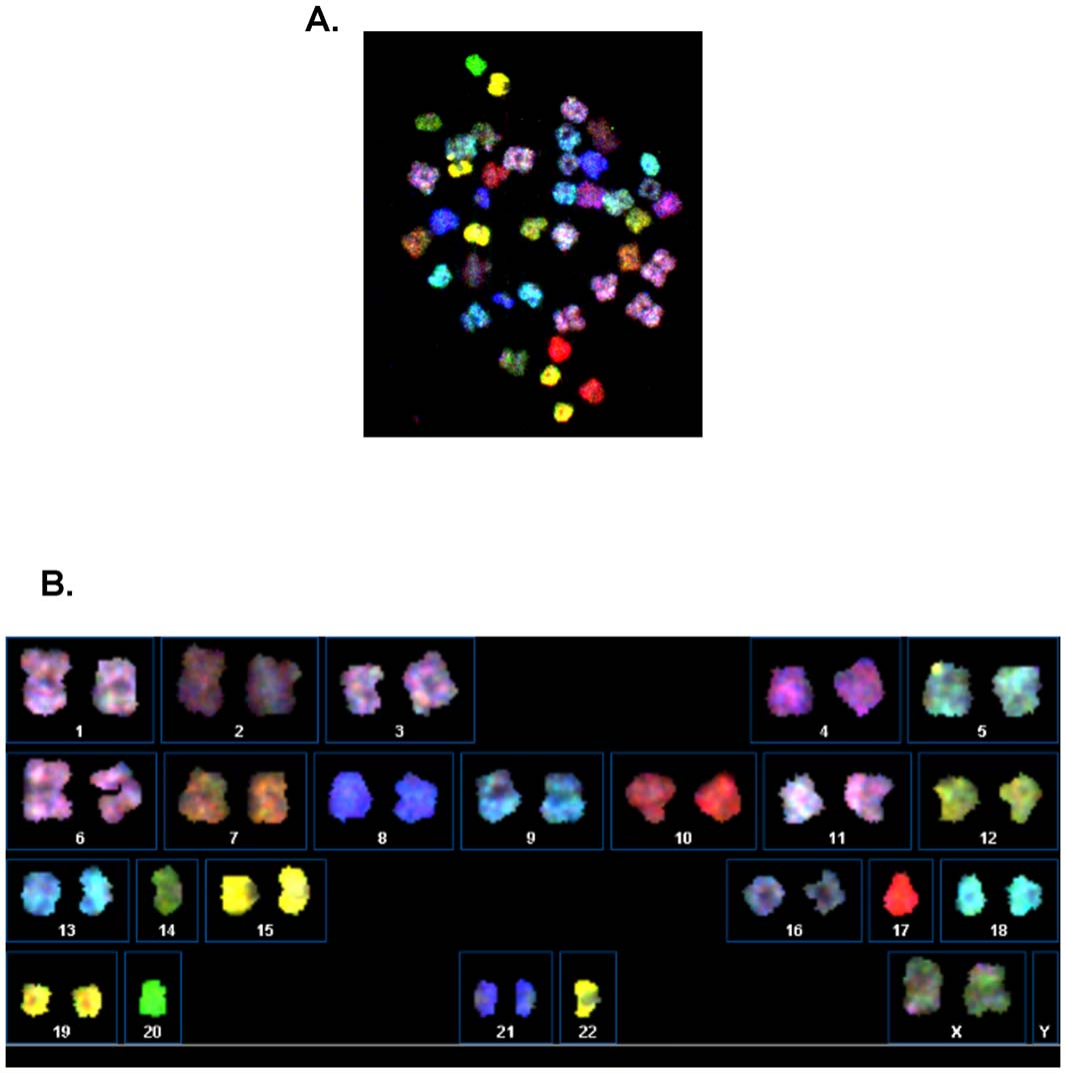
Representative SKY analysis exhibiting mosaic aneuploidy. A) Example of a metaphase spread obtained from H7 cells analyzed by SKY. B) Karyotype table from the spread shown in A demonstrates that the cell had a karyotype of 42, XX, −14, −17, −20, −22.

Since the chromosome counts for aneuploid hPSCs appear as a distribution, it is unlikely that specific clonal karyotypes are being generated. To rule out this possibility, SKY analysis was pursued on two of the hESC lines, H7 and H9, as compared to normal human lymphocytes controls [19]. Forty metaphase chromosome spreads from each sample were analyzed by two independent observers using SKY. Consistent with the results from chromosome counts, approximately 20–30% of the cells from each hESC line were mosaically aneuploid. A representative H7 chromosome spread hybridized with SKY paint (Figure 3A) and its karyotype (Figure 3B) identifies this cell as aneuploid with a karyotype of 42, XX, −14, −17, −20, −22. Other observed karyotypes (Table 2) contrast with an absence of aneuploidy in a lymphocyte sample of 40 spreads analyzed by SKY. These data suggest that chromosome gain and/or loss in mosaically aneuploid hPSCs occurs in a stochastic manner.

**Table 2:** SKY analysis suggests that mosaic aneuploidy is due to stochastic gain and loss of chromosomes.

Cell line	Karyotype
H9 p37	72.5% 46, XX
	2.5% 43, XX, −2, −4, −7, +11, −14
	2.5% 45, XX, −21
	2.5% 46, XX, +3, −10
	2.5% 43, XX, −10, −19, −21
	2.5% 41, XX, −1, −3, −7, −16, −17
	2.5% 46, XX, +5, −12
	2.5% 44, XX, −5, −17
	2.5% 45, XX, −20
	2.5% 43, XX, −11, −15, −16
	2.5% 42, XX, −13, −19, −20, −21
	2.5% 45, XX, −19
H7 p43	80% 46, XX
	2.5% 39, X, −5, −11, −12,−14, −20, −22, −X
	2.5% 45, X
	2.5% 45, XX, −2
	2.5% 42, XX, −14, −17, −20, −22
	2.5% 43, XX, −17, −21, −22
	2.5% 44, XX, −16, −22
	2.5% 42, XX, −6, −11, −14, −17
	2.5% 44, XX, −13, −21
lymphocytes	100% 46, XY

Detailed karyotypes obtained from SKY analysis of H7, H9, and normal lymphocytes. Only numerical aneuploidies are shown, some of which included simultaneous chromosomal gains and losses. Forty chromosome spreads were analyzed for each cell type. There is no statistically significant trend in individual chromosome loss or gain. Individual chromosomes were lost and/or gained at rates of 0–4%, consistent with rates obtained from chromosome counts and FISH analysis.

### FISH confirms that hESC lines exhibit pervasive mosaic aneuploidy

**Figure 4:**
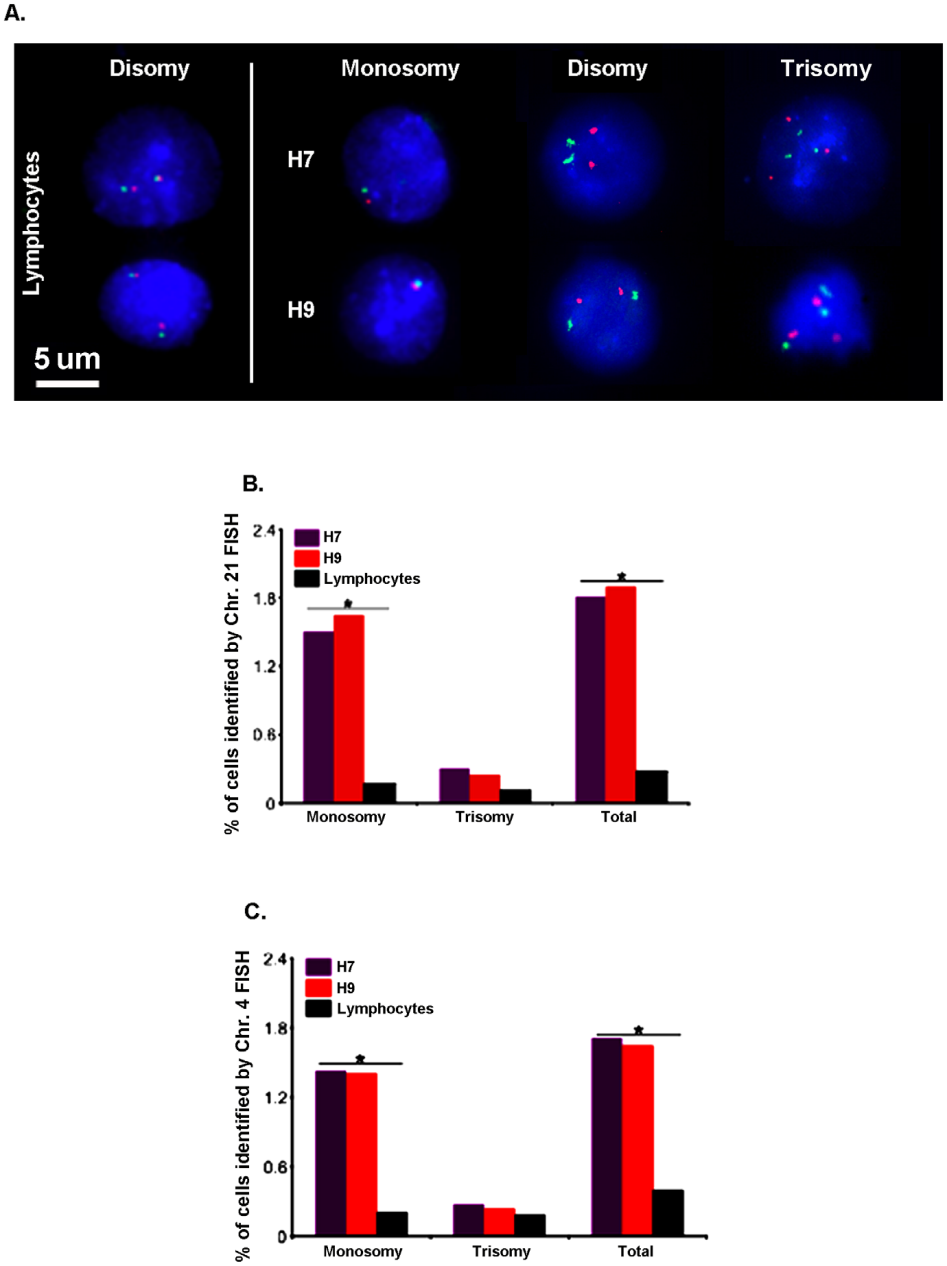
FISH analysis of interphase/non-mitotic hESCs and lymphocytes. A) Nuclei hybridized with dual point probes (red and green) for chromosome 21 and stained with DAPI (blue). Representative examples of monosomic, disomic and trisomic H7 and H9 nuclei, as well as two disomic lymphocyte nuclei are shown. B) Interphase/non-mitotic FISH analysis of H7, H9, and lymphocytes identified a statistically significant 6–7 fold increase in levels of chromosome 21 aneuploidy in hESC lines compared to lymphocytes (*p<0.001, χ^2^). Note that this dual point probe combination has error rates of <0.01%. C) Similar results were obtained for chromosome 4.

To confirm these findings under conditions in which chromosomes could not be artifactually gained or lost, interphase cells from each hESC line were assessed by FISH for autosomal aneuploidy using dual (red and green) point probes against defined loci on chromosome 21 (Figure 4 A–B) and chromosome 4 (Figure 4C). Analyses of at least 3,500 nuclei per sample revealed chromosome 21 and chromosome 4 aneuploidy levels of ∼0.3% in the control lymphocyte sample, consistent with the absence of aneuploid cells detected by SKY using smaller (N = 40) samples. By contrast, ∼1.5–2% of cells from each hESC line were aneusomic for chromosome 21 (Figure 4B) or chromosome 4 (Figure 4C). If extrapolated for the remaining paired chromosomes, the resultant level of mosaic aneuploidy revealed by FISH is consistent with both chromosome counts revealed by DAPI and the metaphase SKY analyses. The difference in aneuploidy levels between the hESC lines and lymphocytes was statistically significant (p<0.001, χ^2^). In addition to the hESC data presented here, mosaic aneuploidy was also observed in mouse ESC (mESC) lines, including E14 and R1, consistent with prior reports on the presence of chromosomal aneuploidy in mESCs [47], [48] (data not shown). Thus, normal PSC lines consist not only of euploid cells, but also contain significant populations of previously unrecognized or unappreciated mosaic aneuploid cells.

## Discussion

The combined use of chromosome counting, SKY, and FISH on multiple hPSC lines, compared to lymphocyte controls, demonstrates that mosaic aneuploidy is not an artifact of cell preparation or culture. Mosaic aneuploidy levels were not affected by culture technique, passage number, laboratory or investigator. In no case were 100% euploid hPSC lines ever observed, with mosaic aneuploidy levels typically ranging between 18–35%. Importantly, mosaic aneuploidy appears to be stochastic, suggesting that it does not confer any selective growth advantage on the cells. This is in contrast to constitutive aneuploidies, which have been frequently reported in late passage and culture adapted hPSC lines [45], [46], [49], [50], [51], [52]. Given the fact that constitutive aneuploidies typically consist of chromosomal gains (chromosome 12, 17, 1, and sometimes X) and have been associated with particular culture or passaging conditions, mosaic aneuploidy represents a distinct karyotypic phenomenon associated with normal PSC lines and is likely an intrinsic characteristic of normal stem cell populations, given that it is seen in normal neural progenitor cells as well as primary cells from the inner cell mass of blastocysts [18], [25], [26], [27], [28].

These data are in stark contrast with previously published reports and patents, which describe hPSCs as 100% euploid [1], [32], [33]. In addition, when H9s from passage 68 shown in Figure 2A were karyotyped at WiCell, the report stated that the cells are “46, XX” and “No abnormalities were detected at the stated band level of resolution” (Supplemental Figure S1). The reason for these differences is unclear but may reflect a conventional bias against the most common form of mosaic aneuploidy in hPSCs, hypoploidy, that is due to chromosome loss. In cytogenetic manuals, this type of aneuploidy is typically dismissed as a technical artifact with the reasoning being that during preparation of the metaphase chromosome spread, individual chromosomes drifted away from the rest of the metaphase spread, thus making the spread hypoploid. In fact, the AGT Cytogenetics Laboratory Manual dictates that “if fewer than 45 chromosomes are present in the metaphase [spread], it can be assumed that some have become lost in the processing and that the metaphase spread is unsuitable for analysis” [34]. The interpretation that hypoploidy is artifactual is pervasive in reports utilizing cytogenetics where hypoploidy is encountered [34], [35], [36]. Given that similar aneuploidy levels were identified using both chromosome counts, SKY and most importantly FISH – which utilizes intact nuclei without condensed chromosomes – along with consistent euploid detection in control lymphocytes, mosaic aneuploidy in hPSCs is not due to a technical artifact.

Cell cycle check-points in PSCs have been studied extensively and may suggest a potential mechanism for the generation of mosaic aneuploidy. PSCs and adult stem cells show cell cycle progression that is distinct from committed, mitotic cells. Instead of a typically long G0/G1 phase, stem cells have almost no G0/G1 phase [26], [53], [54], suggesting that G1 checkpoints may be bypassed. PSCs also appear to tolerate disruption of normal mitotic spindle checkpoints [26], [55] that would normally result in apoptosis. Thus, aneuploidy is better tolerated in PSCs. Another cell cycle check-point that is lax in PSCs is the decatenation check point – which is intended to prevent mitosis when chromosomes become entangled [56]. When PSCs undergo mitosis in the absence of the decatenation check-point, they divide with tangled chromosomes which frequently leads to aneuploidy. In differentiated cells, the decatenation check-point is active and cells are not allowed to divide with tangled chromosomes, thus preventing aneuploidy. The fact that PSC cell cycle check-points are clearly lax compared to other cells could provide a mechanism for the generation of mosaic aneuploidy and also suggests that the presence of mosaic aneuploidy in these cells is in part a consequence of their intrinsic cell cycle characteristics. In normal, neural progenitor cells, aneuploidy can be generated by well known chromosomal segregation mechanisms that include supernumerary centrosomes, lagging chromosomes, multipolar divisions and non-disjunction [57], all of which may also contribute to PSC mosaic aneuploidy.

The existence of mosaic aneuploidy intrinsic to PSC lines that produces a karyotypically heterogenous, diverse stem cell line complements the well-documented phenotypic and functional heterogeneity observed in PSCs. We speculate that the genomic diversity produced by mosaic aneuploidy accounts for or contributes to the previously reported alterations in gene expression and downstream phenotypes [10], [11], [12], [13], [14], [15]. It has been suggested that the heterogeneity in PSC cultures allows them to both respond to differentiation inducing factors and to retain their ability to self renew [12]. This may provide subpopulations within a given PSC line with growth or survival advantages, depending on the environmental conditions – in culture or in vivo – encountered by the population as a whole. These interactions may be both cell autonomous as well as non-cell autonomous, which could endow PSC lines with a “fitness” that promotes a desirable cellular endpoint, ranging from pluripotency to appropriate differentiation.

**Figure 5:**
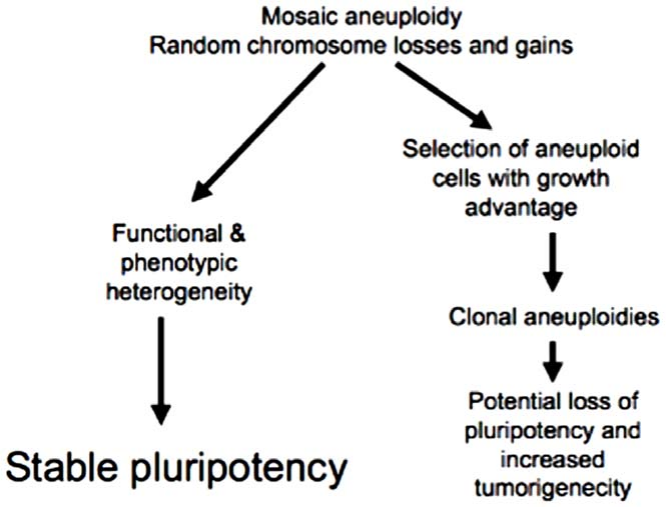
Model of the effects of mosaic aneuploidy on hPSC cultures. Mosaic aneuploidy may have both beneficial and detrimental effects - on the one hand, it may generate functional and phenotypic heterogeneity within stem cell populations leading to stable pluripotency. On the negative side, it may generate cells with a selective advantage that may be clonally expanded leading to a decrease in pluripotency and potential tumorigenicity.

Constitutive aneuploidies have historically been associated with carcinogenesis and it is possible, even probable, that some rare karyotypes present within *normal* mosaically aneuploid hPSC lines could have increased growth potential under defined conditions. We speculate that previously reported, clonally aneuploid and hyperploid hESC lines [33], [45], [51], [58] arose initially from normal mosaic aneuploid hESC lines that contained these rare karyotypes (e.g., gain of chromosome 12). Mosaic aneuploidy may therefore be both beneficial by sustaining pluripotency and differentiation potential, and also detrimental by generating karyotypes that have a growth advantage and carcinogenic potential (Figure 5). This issue is especially important for hPSC therapeutic approaches that involve transplantation of an entire population of a given hPSC line and/or its derivatives, since it will consist of cells with both advantageous and disadvantageous genotypes. Further consideration of mosaic aneuploidy – and other genomic changes that can produce diversity, such as copy number variants [59], [60] – could help to explain PSC heterogeneity and improve both the efficacy and safety of future stem cell uses.

## Supporting Information

Figure S1 H9s from passage 68 (same cells as those used in Figure 2A) were karyotyped at WiCell. Their report indicates that the cells are “46,XX” and “No abnormalities were detected at the stated band level of resolution.”
(PDF)

## References

[1] Thomson JA, Itskovitz-Eldor J, Shapiro SS, Waknitz MA, Swiergiel JJ (1998). Embryonic stem cell lines derived from human blastocysts.. Science.

[2] Takahashi K, Yamanaka S (2006). Induction of pluripotent stem cells from mouse embryonic and adult fibroblast cultures by defined factors.. Cell.

[3] Muller FJ, Laurent LC, Kostka D, Ulitsky I, Williams R (2008). Regulatory networks define phenotypic classes of human stem cell lines.. Nature.

[4] Efroni S, Duttagupta R, Cheng J, Dehghani H, Hoeppner DJ (2008). Global transcription in pluripotent embryonic stem cells.. Cell Stem Cell.

[5] Bibikova M, Chudin E, Wu B, Zhou L, Garcia EW (2006). Human embryonic stem cells have a unique epigenetic signature.. Genome Res.

[6] Laurent LC, Chen J, Ulitsky I, Mueller FJ, Lu C (2008). Comprehensive microRNA profiling reveals a unique human embryonic stem cell signature dominated by a single seed sequence.. Stem Cells.

[7] Lister R, Pelizzola M, Dowen RH, Hawkins RD, Hon G (2009). Human DNA methylomes at base resolution show widespread epigenomic differences.. Nature.

[8] Laurent L, Wong E, Li G, Huynh T, Tsirigos A Dynamic changes in the human methylome during differentiation.. Genome Res.

[9] Mack GS ReNeuron and StemCells get green light for neural stem cell trials.. Nat Biotechnol.

[10] Hough SR, Laslett AL, Grimmond SB, Kolle G, Pera MF (2009). A continuum of cell states spans pluripotency and lineage commitment in human embryonic stem cells.. PLoS One.

[11] Chang HH, Hemberg M, Barahona M, Ingber DE, Huang S (2008). Transcriptome-wide noise controls lineage choice in mammalian progenitor cells.. Nature.

[12] Graf T, Stadtfeld M (2008). Heterogeneity of embryonic and adult stem cells.. Cell Stem Cell.

[13] Chambers I, Silva J, Colby D, Nichols J, Nijmeijer B (2007). Nanog safeguards pluripotency and mediates germline development.. Nature.

[14] Hayashi K, Lopes SM, Tang F, Surani MA (2008). Dynamic equilibrium and heterogeneity of mouse pluripotent stem cells with distinct functional and epigenetic states.. Cell Stem Cell.

[15] Singh AM, Hamazaki T, Hankowski KE, Terada N (2007). A heterogeneous expression pattern for Nanog in embryonic stem cells.. Stem Cells.

[16] Chin MH, Mason MJ, Xie W, Volinia S, Singer M (2009). Induced pluripotent stem cells and embryonic stem cells are distinguished by gene expression signatures.. Cell Stem Cell.

[17] Tanasijevic B, Dai B, Ezashi T, Livingston K, Roberts RM (2009). Progressive accumulation of epigenetic heterogeneity during human ES cell culture.. Epigenetics.

[18] Rehen SK, McConnell MJ, Kaushal D, Kingsbury MA, Yang AH (2001). Chromosomal variation in neurons of the developing and adult mammalian nervous system.. Proc Natl Acad Sci U S A.

[19] Rehen SK, Yung YC, McCreight MP, Kaushal D, Yang AH (2005). Constitutional aneuploidy in the normal human brain.. J Neurosci.

[20] Forni PE, Scuoppo C, Imayoshi I, Taulli R, Dastru W (2006). High levels of Cre expression in neuronal progenitors cause defects in brain development leading to microencephaly and hydrocephaly.. J Neurosci.

[21] Yurov YB, Iourov IY, Monakhov VV, Soloviev IV, Vostrikov VM (2005). The variation of aneuploidy frequency in the developing and adult human brain revealed by an interphase FISH study.. J Histochem Cytochem.

[22] Rajendran RS, Zupanc MM, Losche A, Westra J, Chun J (2007). Numerical chromosome variation and mitotic segregation defects in the adult brain of teleost fish.. Dev Neurobiol.

[23] Kingsbury MA, Friedman B, McConnell MJ, Rehen SK, Yang AH (2005). Aneuploid neurons are functionally active and integrated into brain circuitry.. Proc Natl Acad Sci U S A.

[24] Kaushal D, Contos JJ, Treuner K, Yang AH, Kingsbury MA (2003). Alteration of gene expression by chromosome loss in the postnatal mouse brain.. J Neurosci.

[25] Vanneste E, Voet T, Le Caignec C, Ampe M, Konings P (2009). Chromosome instability is common in human cleavage-stage embryos.. Nat Med.

[26] Ambartsumyan G, Clark AT (2008). Aneuploidy and early human embryo development.. Hum Mol Genet.

[27] Frumkin T, Malcov M, Yaron Y, Ben-Yosef D (2008). Elucidating the origin of chromosomal aberrations in IVF embryos by preimplantation genetic analysis.. Mol Cell Endocrinol.

[28] Lavon N, Narwani K, Golan-Lev T, Buehler N, Hill D (2008). Derivation of euploid human embryonic stem cells from aneuploid embryos.. Stem Cells.

[29] Dupont C, Segars J, DeCherney A, Bavister BD, Armant DR Incidence of chromosomal mosaicism in morphologically normal nonhuman primate preimplantation embryos.. Fertil Steril.

[30] Wilton L (2002). Preimplantation genetic diagnosis for aneuploidy screening in early human embryos: a review.. Prenat Diagn.

[31] Munne S, Weier HU, Grifo J, Cohen J (1994). Chromosome mosaicism in human embryos.. Biol Reprod.

[32] Thomson JA (2001). Primate Embryonic Stem Cells..

[33] Cowan CA, Klimanskaya I, McMahon J, Atienza J, Witmyer J (2004). Derivation of embryonic stem-cell lines from human blastocysts.. N Engl J Med.

[34] Barch M, Knutsen T, Spurbek J (1997). The AGT Cytogenetics Laboratory Manual.

[35] Zheng P, Dean J (2009). Role of Filia, a maternal effect gene, in maintaining euploidy during cleavage-stage mouse embryogenesis.. Proc Natl Acad Sci U S A.

[36] Meisner LF, Johnson JA (2008). Protocols for cytogenetic studies of human embryonic stem cells.. Methods.

[37] Mitalipova M, Calhoun J, Shin S, Wininger D, Schulz T (2003). Human embryonic stem cell lines derived from discarded embryos.. Stem Cells.

[38] D'Amour KA, Agulnick AD, Eliazer S, Kelly OG, Kroon E (2005). Efficient differentiation of human embryonic stem cells to definitive endoderm.. Nat Biotechnol.

[39] Peterson S, Rehen S, Westra W, Yung Y, Chun J, Loring J, Wesselschmidt RL, Schwartz PH (2007). Spectral Karyotyping and Fluorescent in situ Hybridization.. Human Stem Cell Manual A Laboratory Guide.

[40] Schrock E, du Manoir S, Veldman T, Schoell B, Wienberg J (1996). Multicolor spectral karyotyping of human chromosomes.. Science.

[41] Takahashi K, Tanabe K, Ohnuki M, Narita M, Ichisaka T (2007). Induction of pluripotent stem cells from adult human fibroblasts by defined factors.. Cell.

[42] Meissner A, Wernig M, Jaenisch R (2007). Direct reprogramming of genetically unmodified fibroblasts into pluripotent stem cells.. Nat Biotechnol.

[43] Yu J, Vodyanik MA, Smuga-Otto K, Antosiewicz-Bourget J, Frane JL (2007). Induced pluripotent stem cell lines derived from human somatic cells.. Science.

[44] Wernig M, Meissner A, Foreman R, Brambrink T, Ku M (2007). In vitro reprogramming of fibroblasts into a pluripotent ES-cell-like state.. Nature.

[45] Draper JS, Smith K, Gokhale P, Moore HD, Maltby E (2004). Recurrent gain of chromosomes 17q and 12 in cultured human embryonic stem cells.. Nat Biotechnol.

[46] Spits C, Mateizel I, Geens M, Mertzanidou A, Staessen C (2008). Recurrent chromosomal abnormalities in human embryonic stem cells.. Nat Biotechnol.

[47] Eggan K, Rode A, Jentsch I, Samuel C, Hennek T (2002). Male and female mice derived from the same embryonic stem cell clone by tetraploid embryo complementation.. Nat Biotechnol.

[48] Longo L, Bygrave A, Grosveld FG, Pandolfi PP (1997). The chromosome make-up of mouse embryonic stem cells is predictive of somatic and germ cell chimaerism.. Transgenic Res.

[49] Rosler ES, Fisk GJ, Ares X, Irving J, Miura T (2004). Long-term culture of human embryonic stem cells in feeder-free conditions.. Dev Dyn.

[50] Pera MF (2004). Unnatural selection of cultured human ES cells?. Nat Biotechnol.

[51] Enver T, Soneji S, Joshi C, Brown J, Iborra F (2005). Cellular differentiation hierarchies in normal and culture-adapted human embryonic stem cells.. Hum Mol Genet.

[52] Imreh MP, Gertow K, Cedervall J, Unger C, Holmberg K (2006). In vitro culture conditions favoring selection of chromosomal abnormalities in human ES cells.. J Cell Biochem.

[53] Becker KA, Stein JL, Lian JB, van Wijnen AJ, Stein GS Human embryonic stem cells are pre-mitotically committed to self-renewal and acquire a lengthened G1 phase upon lineage programming.. J Cell Physiol.

[54] Carpenter MK, Rosler E, Rao MS (2003). Characterization and differentiation of human embryonic stem cells.. Cloning Stem Cells.

[55] Mantel C, Guo Y, Lee MR, Kim MK, Han MK (2007). Checkpoint-apoptosis uncoupling in human and mouse embryonic stem cells: a source of karyotpic instability.. Blood.

[56] Damelin M, Sun YE, Sodja VB, Bestor TH (2005). Decatenation checkpoint deficiency in stem and progenitor cells.. Cancer Cell.

[57] Yang AH, Kaushal D, Rehen SK, Kriedt K, Kingsbury MA (2003). Chromosome segregation defects contribute to aneuploidy in normal neural progenitor cells.. J Neurosci.

[58] Mitalipova MM, Rao RR, Hoyer DM, Johnson JA, Meisner LF (2005). Preserving the genetic integrity of human embryonic stem cells.. Nat Biotechnol.

[59] Narva E, Autio R, Rahkonen N, Kong L, Harrison N High-resolution DNA analysis of human embryonic stem cell lines reveals culture-induced copy number changes and loss of heterozygosity.. Nat Biotechnol.

[60] Laurent LC, Ulitsky I, Slavin I, Tran H, Schork A Dynamic changes in the copy number of pluripotency and cell proliferation genes in human ESCs and iPSCs during reprogramming and time in culture.. Cell Stem Cell.

