# Supplementary material for: Normal Human Pluripotent Stem Cell Lines Exhibit Pervasive Mosaic Aneuploidy
Source: PLoS One. 2011 Aug 16;6(8):e23018. doi: 10.1371/journal.pone.0023018 (PMC3156708; doi:10.1371/journal.pone.0023018)
Supplement: Figure S1 — H9s from passage 68 (same cells as those used in Figure 2A) were karyotyped at WiCell. Their report indicates that the cells are “46,XX” and “No abnormalities were detected at the stated band level of resolution.” (PDF) [file pone.0023018.s001.pdf]

Peterson - Supplemental Figure 1

WiCell Cytogenetics Report: 001680-041010

Report Date: April 13, 2010

Case Details:

Cell Line: WA09

Passage #: 68

Date Completed: 4/13/2010

Cell Line Gender: Female

Investigator: Suzanne Peterson

Specimen: hESC on MEF feeder

Date of Sample: 4/8/2010

Tests,Reason for: Routine Testing

Results: 46,XX
Completed by Kim Leonhard, CG(ASCP), on 4/13/2010

Reviewed and interpreted by Karen Dyer Montgomery, PhD, FACMG, on 4/13/2010

Interpretation: No abnormalities were detected at the stated band level of resolution.

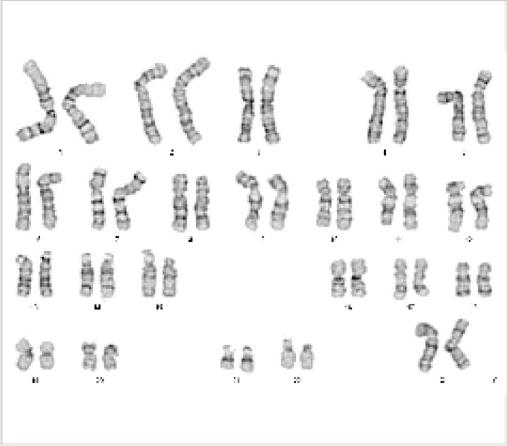

Cell: S01-01

Slide: A-2

Slide Type: Karyotyping

# of Cells Counted: 20

# of Cells Karyotyped: 4

# of Cells Analyzed: 8

Band Level: 425-575

Results Transmitted by Fax / Email / Post
Sent By:_______________________
QC Review By: _______________________

Date:_______________________
Sent To:_______________________
Results Recorded: _______________
